# What advice should we give our patients to preserve their fertility and avoid needing oocyte donation in the future? - A Social Fertility Preservation program

**DOI:** 10.5935/1518-0557.20180088

**Published:** 2019

**Authors:** Luis Guzman, Naomi Inoue, Denisse Núñez, Jazmin Meza, Pedro Bendezu, Pilar Pino, Jimmy Portella, Luis Noriega-Portella, Luis Noriega-Hoces

**Affiliations:** 1 PRANOR Laboratorio. Grupo de Reproducción Asistida. Av. Monterrico 1045. Urb El Derby de Monterrico Lima 55, Perú; 2 Clínica Concebir. Calle Los Olivos 364. San Isidro. Lima 31. Perú

**Keywords:** fertility preservation, social freezing, oocyte vitrification, oocyte donation

## Abstract

**Objective:**

To describe our fertility preservation program focusing on the number of oocytes vitrified by age.

**Methods:**

From January 2015 to December 2016, 686 oocyte vitrification cycles were performed in our units for the social fertility preservation program. In total, 288 were donors who donated their oocytes for our oocyte-banking program, and 398 were patients who underwent elective fertility preservation.

**Results:**

The mean numbers of COCs retrieved and vitrified oocytes were similar among the donor cycles (women under 30 years). In those patients over 36 years of age the mean numbers of COCs retrieved and vitrified oocytes were significantly lower. We also estimated the association between age and cancelation rates. Odd ratios (OR) for total cancelation was calculated between patients of 31-35 years and 41-45 years; the OR was 5.17 (95% CI 1.89 - 14.17) and increased up to 25.67 (95% CI 5.01 - 131.42) between patients 31-35 y and those older than 45 years. No differences were found between patients of 31-35 years and 36-40 years. The OR for total cancellation increased 3.83 (95% CI 2.06 - 7.11) and 19.00 (95% CI 4.56 - 79.11) between women 36-40 years and 41-45 years, and those older than 45 years, respectively. Finally, the oocyte survival rate in patients under 36 years of age was similar to that of our donor program (94% *vs.* 95%).

**Conclusions:**

Based on this study, we encouraged our patients under than 36 years of age to preserve their fertility for the future.

## INTRODUCTION

Women have been delaying motherhood to later than their forties, although their oocyte quality decrease and aneuploidy rates increase by age. For these women, there are two options: (1) Oocyte banking, this strategy involves patients undergoing consecutive stimulation cycles that would allow banking of a large amount of oocytes or embryos and (2) oocyte donation, which involves a healthy volunteer donating her oocytes for assisted reproduction treatments. The first pregnancy achieved after oocyte donation (OD) was reported in 1984 (Lutjen *et al*., 1984). Since then, thousands of pregnancies have been achieved worldwide. Currently, almost all IVF programs include cycles with OD; this treatment has become increasingly more accepted, since live births increase dramatically, up to 15-fold, in women aged ≥40 (Martin *et al.*, 2007).

At the present, OD is indicated in patients with premature ovarian failure, advanced maternal age (AMA), secondary infertility, multiple IVF failure treatments (Pados *et al*., 1994), maternally inherited genetic diseases and women who do not produce euploid embryos (Wang *et al*., 2010). However, the most frequent reasons to undergo OD is AMA, as a result of delaying child bearing (Paulson *et al*., 2002). Although OD is considered an effective treatment, whether with fresh or vitrified oocytes (García *et al*., 2011), higher perinatal complications rates have been reported in OD pregnancies, such as pregnancy-induced hypertension, hemorrhage in the first quarter (Moffett & Loke, 2006) and prematurity (Michalas *et al*., 1996). Other aspects in heterologous OD are the special immunological features conceptus tolerance, because in naturally conceived pregnancies, half of the HLA is from the mother and the other complement is allogenic (from the father). Nevertheless, pregnancies in OD treatments are allogenic, establishing a conceptus tolerance (van der Hoorn *et al*., 2010).

On the other hand, the social implication for accepting a genetic background from a donor is the main physiological reason from the partners to refuse this treatment. A social fertility program provides patients with the option to conceive their own genetically-linked child in the future. This is possible thanks to the advances in vitrification, which has shown higher surviving rates (above 93%) (Cobo *et al*., 2015), and comparable clinical outcomes from vitrified-warmed oocytes compared to fresh IVF cycles (Cobo & Diaz, 2011).

This paper describes our fertility preservation program focusing on the number of vitrified oocytes by age.

## MATERIALS AND METHODS

### Patients

From January 2015 to December 2016, in total 686 cycles of social fertility preservation were been performed in our units. Two hundred eighty eight were oocyte donation cycles and three hundred ninety eight were patients who want to preserve their fertility for the future.

### Ovarian stimulation

All patients received ovarian stimulation through a GnRH antagonist protocol, using a combination either of recombinant follicle-stimulating hormone plus recombinant luteinizing hormone (r-FSH and r-FSH/r-LH; Gonal^®^ and Pergoveris^®^), or highly purified follicle stimulant hormone plus highly purified human menopausal gonadotropin (hp-FSH and hp-HMG; Bravelle^®^ and Menopur^®^) from day 2 or 3 of the menstrual cycle. We administered daily doses of 0.25 mg GnRH antagonist (cetrorelix acetate, Cetrotide^®^) from day 6 of stimulation until the day of triggering, when at least 2 follicles reached 18 mm. We administered 250 IU of recombinant hCG (r-hCG, Ovidrel^®^) or Triptorelin 0.2 mg (Gonapeptyl daily^®^) to trigger the LH surge. We used gonadotropin at a dose range between 225-300 IU. We based the decision regarding dose to use based on the antral follicles count, the antimullerian hormone value and patient age. We used transvaginal ultrasound and serum estradiol to control ovarian stimulation. Transvaginal oocyte retrieval was performed at 35-36 hours after triggering the LH surge.

### Oocyte Collection

Oocyte cumulus complexes (COC) were collected in flushing media (Irvine); the COCs were identified under a stereomicroscope (Olympus SX-16) and collected in global total with hepes. The excess of cumulus cells were cut and the oocytes were placed in a global fertilization media.

### Oocyte Vitrification

The oocytes were denuded 2 hours after retrieval. After nuclear maturity evaluation, only the MII oocytes were selected for immediate vitrification. All the vitrification and warming solutions were prepared in modified Eagle medium HEPES-buffered media and were obtained from Kitazato.

The cryotop method employed for oocyte vitrification was that described by Kuwayama *et al*. (2005), with minimal modifications. The oocytes were equilibrated at room temperature for 15 min in 7.5% (v/ v) ethylene glycol (EG) + 7.5% dimethyl sulfoxide (DMSO) in a TCM199 medium +20% synthetic serum substitute (SSS), referred to as 'equilibrium solution' (ES). As in most cases, more than eight oocytes were equilibrated at the same time, they were checked for recovery of their initial shape at 12 min; if possible, they were subjected to a vitrification step at this point. They were then placed in a 'VS-vitrification solution' that was the same as the ES, except that the concentrations were 15% EG + 15% DMSO + 0.5 M sucrose. After 1 min in this solution, the oocytes were placed on the cryotop strip and immediately submerged in liquid nitrogen (LN). No more than four oocytes per cryotop were loaded.

### Oocyte thawing

We warmed the oocytes following the manufacture's recommendations - briefly a solution of 1 M sucrose or trehalose was warmed at 37ºC. The oocytes were placed rapidly into this solution for 1 minute. Then they were placed in a half-diluted solution for 3 minutes. Finally, the oocytes were washed twice for 5 min and 1 minute respectively. We then placed the oocytes in regular culture conditions for 2 hours until ICSI.

### Statistical analysis

We used the Fisher exact test for categorical variables. The continuous variables did not show a normal distribution, and therefore we used the Mann-Whitney U-test. We calculated the Spearman rank correlation coefficients and corresponding *p*-values. Subsequently, a stepwise regression analysis was performed to identify which subset of variables correlated independently to clinical pregnancy. 

## RESULTS

From January of 2015 through December of 2016, we had 686 cycles of oocyte vitrification, including patients and donors. No complications were observed in any of the stimulation cycles. Overall, 8005 cumulus oocytes complexes (COCs) were retrieved, and 6382 oocytes were vitrified. The maturation rate was 80% in the groups of donors and patients.

The mean numbers of COCs retrieved in each donor group (by age) are shown in Table 1A. In the same group, the maturation rate was similar between age sub-groups 81% (1361/1683); 81% (1912/2371) and 77% (1074/1402) in donors <21y; 21-25y and 26-30y, respectively. Table 1B shows the mean numbers of oocytes retrieved and vitrified in donors. The maturation rates was also similar between the study sub-groups (67% in <21y; 77% in 21-25y; 84% in 26-30y; 77% in 31-35; 81% in 36-40y; 80% in 41-45y and >92% in patients older than 45y).

**Table 1:** Numbers of retrieved and vitrified oocytes stratified by age

A. Donors								
Group	Age	Nº	Doses of gonadotropins	Nº oocyte retrieval	Nº of oocyte MII	Nº of oocyte GVBD	Nº of oocyte GV	Nº of atretic oocyte
DONOR	<21	96	2087±369	17.53±8.95	14.18±7.68	0.84±1.11	1.97±2.74	0.50±0.79
	21-25	121	2062±369	19.60±13.10	15.80±10.77	0.83±1.22	2.24±3.29	0.50±0.85
	26-30	71	2056±434	19.75±12.70	15.13±9.74	0.83±1.11	2.85±3.37	1.00±2.74
B. Patients								
Group	Age	Nº	Doses of gonadotropins	Nº oocyte retrieval	Nº of oocyte MII	Nº of oocyte GVBD	Nº of oocyte GV	Nº of atretic oocyte
PATIENT	<21	2	2025±250	12.00±7.07	8.00±.24	1.50±0.71	2.00±1.41	0.50±0.71
	21-25	15	2133±342	17.80±11.92	13.60±8.96	0.93±1.10	2.80±4.74	0.47±0.74
	26-30	19	2300±156	12.79±7.26^a^	10.89±6.91	0.84±1.07	0.79±.92^a^	0.26±0.45
	31-35	60	2371±558	9.60±7.54^a^	7.43±6.05^a^	0.50±0.89	1.33±2.12^a^	0.23±0.62
	36-40	192	2285±841	5.63±5.92^a^^b^^c^	4.54±4.91^a^^b^^c^	0.36±0.70	0.53±1.04^a^^c^	0.17±0.44
	41-45	100	2066±807	3.46±3.72^a^^b^^c^	2.79±3.15^a^^b^^c^	0.18±0.48^a^^b^	0.23±0.57^a^^c^	0.20±0.68
	>45	10	1800±252	1.30±1.25^a^^b^^c^	1.20±1.32^a^^b^^c^	0.00^a^	0.00^a^	0.10±0.32

Oocytes vitrified at the MII stage. MII= metaphase II; GVBD= germinal vesicle breakdown; GV= germinal vesicle. The statistical analysis was performed and should be interpreted as follows:

a statistically different means of the following age-groups 21 - 25 (*p*<0.05)

b with 26 - 30 (*p*<0.05)

c with 31 - 35 (*p*<0.05).

We calculated and classified the cancelation rate per schedule oocyte pick-up according to two reasons: either the cycles did not have retrieved COCs or because no mature oocytes were obtained. These results are depicted in Table 2.

**Table 2:** Cancellation rates per cycle without oocytes retrieved and without any mature oocytes. Data is presented by age groups

Group		Cycles without retrieved oocytes (%)	Cycles without matured oocytes (%)	Cancelation rate per schedule oocyte retrieval (%)
DONOR	<21	1 (1/96)	0 (0/95)	1(1/96)
	21-25	2 (2/121)	0(0/119)	2 (2/121)
	26-30	0(0/71)	0 (0/71)	0(0/71)
PATIENT	<21	0 (0/2)	0 (0/2)	0 (0/2)
	21-25	0 (0/15)	0 (0/15)	0 (0/15)
	26-30	0 (0/19)	0 0 (0/19)	0 (0/19)
	31-35	5 (3/60)	4 (2/55)	8 (5/60)
	36-40	8 (15/192)	3(6/177)	11 (21/192)
	41-45	19 (19/100)	16 (13/81)	32 (32/100)
	>45	40 (4/10)	50 (3/6)	70(7/10)

**Figure 1:**
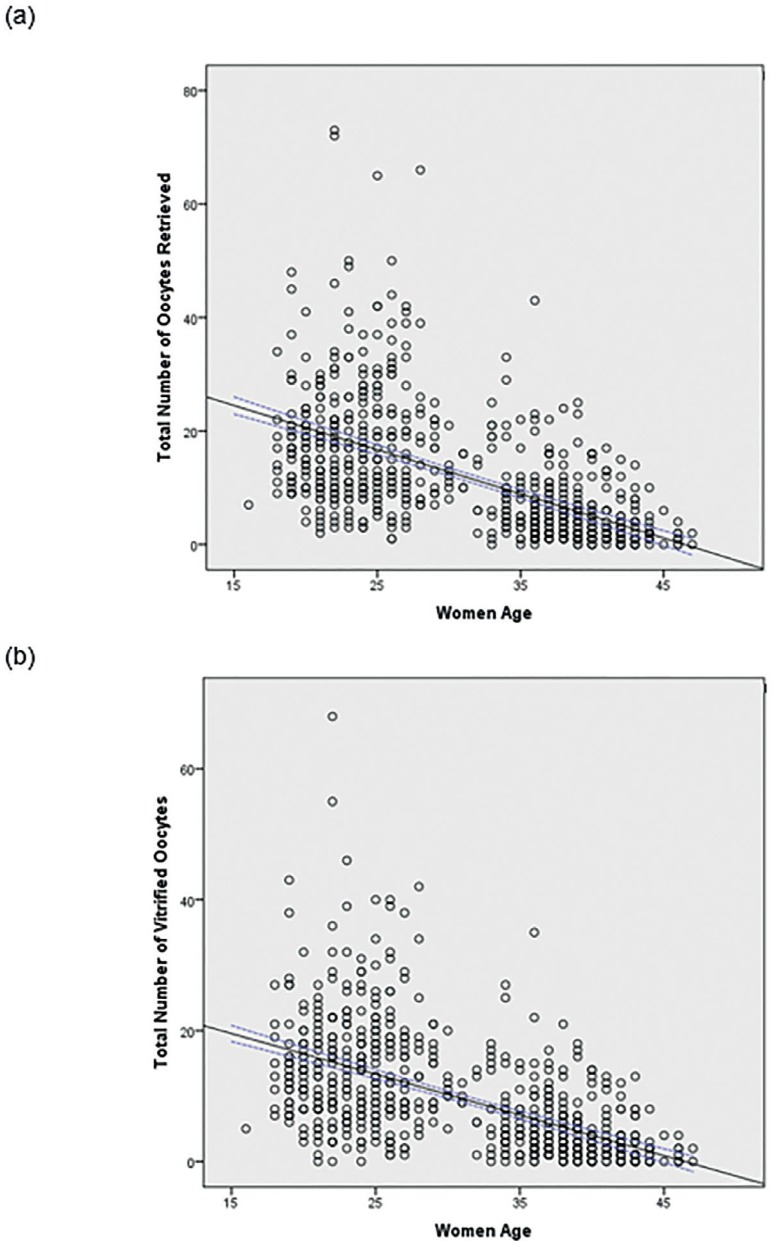
A scatter plot distribution showing the number of oocytes retrieved (a) and number of vitrified oocytes by age (b)

We estimated the association between age and cancelation rates (Figure 1). Odd ratios (OR) for total cancelation rates (either due to cycles without retrieved COCs or any mature oocytes) was calculated between patients of 31-35 years and 41-45 years; OR was 5.17, 95% CI (1.89 - 14.17) (*p*<0.001) and increased up to 25.67 95% CI (5.01 - 131.42) (*p*<0.001) between 31-35y and older than 45 years. Interestingly, we found no differences between patients of 31-35 years and 36-40 years. However, the OR for total cancellation was increased up to 3.83; 95% CI (2.06 - 7.11) (*p*<0.001) and 19.00; 95% CI (4.56 - 79.11) (*p*<0.001) between women among 36-40 years and 41-45 years; older than 45 years, respectively.

**Figure 2:**
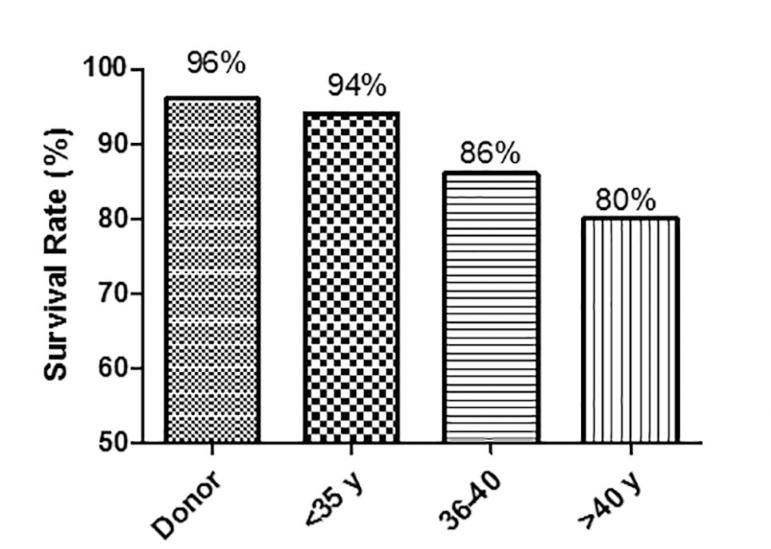
Survival rate of patients who decided to thaw their oocytes in the same period of our study

Finally, we thawed the oocytes and calculated their survival rate according to the oocyte source (donors or patients) and age group; in the donor group, the survival rate was 96%; among patients under 35 years of age, survival was 94%, and it fell to 80% in patients older than 40 years (results are shown on Figure 2).

## DISCUSSION

Social freezing is an interesting topic in assisted reproduction, because of delayed motherhood in the last decades. Reasons for delayed the first pregnancy are complex, including personal, professional or financial difficulties (Mertes & Pennings, 2011). Therefore, every year more women at advance maternal age are coming to the fertility centers for an IVF treatment. However, these patients have a higher change to have recurrently failed IVF cycles. The other suitable alternative is oocyte donor cycles, which involves an extensive counselling, which is usually refused as first treatment option.

Oocyte donation cycles have good clinical outcomes, which can reach up to 80% of clinical pregnancy rates. Nevertheless, there are some safety concerns regarding the oocyte donation program. Although results are not clear, some studies have pointed to a high risk of pre-eclampsia, the mode of delivery and an elevated rate of prematurity (Sauer *et al.*, 1996). Additionally, the insertion of unknown genetically intact fetal cells into the maternal circulation (fetal cell microchimerism) might be also relevant to egg donation pregnancies, because we still do not know whether these circulating fetal cells might play a role in establishing or maintaining tolerance to the conceptus. Furthermore, the consequences of persistent foreign circulating fetal cells for the mother's long-term health are currently unknown. Nonetheless, in one study, allogenenic male fetal cells were shown to persist for up to 9 years in the circulation of healthy post-partum women who conceived using egg donors and delivered male infants (Williams *et al*., 2009). The implications of becoming microchimeric with an unmatched population of fetal progenitor cells are still unknown.

In the last decade, oocyte cryopreservation has changed from slow freezing to oocyte vitrification, which improved the survival rate and maintained oocyte development competence as in fresh oocytes (Kuwayama *et al*., 2005; Vajta & Nagy, 2006). This has allowed clinicians to offers several alternatives, such as oocyte banking for low responder patients (Cobo *et al*., 2012), unavailability of semen sample collection, risk of ovarian hyperstimulation syndrome and even delayed embryo transfer (Herrero *et al*., 2014). Vitrification has also proven to be a useful tool, which is currently used in oncofertility cases, egg donor banks and social fertility programs.

Social fertility programs have been created to enroll women at younger ages. Nevertheless, a study has shown that 63% of patients are cryopreserving their oocytes between 37 and 39 years, and 16.2% were women older than 40 (Cobo *et al*., 2016b). Our results show that 48.2% of the oocyte cryopreservation procedures were performed in women between 36 and 40 years, and 27.6% were patients over 40 years of age. This information brings about a concern regarding our current communication strategy to educate our patients for elective fertility preservation at younger ages.

An interesting study conducted in the USA in 2017 (Milman *et al*., 2017) showed that 87% of women between 21 and 45 years were aware of elective oocyte cryopreservation, but only 0.9% had their oocytes vitrified. Additionally, after knowing or learning about the whole oocyte vitrification procedure, only 21.6% were likely to preserve their fertility for the future. However, another similar study conducted in Belgium, in 2010, showed that 31.5% were willing to preserve their fertility (Stoop *et al*., 2011). Consequently, a tutorial procedure has to be stablished according to each society and country to educate patients, trying to persuade them to vitrify their oocytes at younger ages and avoid oocyte donation in the future.

Oocyte vitrification safety is an important message that must be delivered clearly to our patients. Vitrification safety could be demonstrated based on the prospective randomized studies conducted on sibling oocytes in infertile patients, where fresh and cryopreserved oocytes had comparable clinical outcomes (Cobo *et al.*, 2008; Rienzi *et al*., 2012). Similar outcomes have been shown in donor cycles, demonstrating that vitrification shows comparable results, either using fresh or cryopreserved oocytes (Kim *et al*., 2010). Interestingly, the effects of vitrification are also found in older women, where the efficiency of oocyte vitrification shows results comparable to those from fresh cycles (Cil *et al*., 2013; Doyle *et al*., 2016). Despite decreases in survival rates from 96%, in women younger than 34 years, to 83% in women older than 35 years (Cobo & Garcia-Velasco, 2016a), all these studies confirmed that oocyte vitrification is a safe procedure that does not change pregnancy likelihoods by using fresh or vitrified oocytes; and more importantly, the children conceived from vitrified oocytes have similar perinatal outcomes, suggesting that this procedure is harmless (Cobo *et al*., 2014).

Regarding the safety of how long the oocytes could be maintained in liquid nitrogen, a study has shown that survival rates and developmental competence remained unaltered in a period of 6 months to 5 years (Cobo *et al*., 2015). Additionally, these oocytes were stored in vapor liquid nitrogen tanks, which proves that vapor storage for vitrified samples in very small volumes could be used without concerns (Stoop *et al*., 2012).

Counselling should also include a financial explanation of cost-benefit, which included the total cost of cryopreserving their oocytes per year; the chances of utilizing their oocytes in the future and estimation of storage-time, which could be calculated, based on the vitrification age and the mean aged of patients treated in each fertility center.

In conclusion, and based on our findings, oocyte cryopreservation for social purposes is an interesting alternative for women who, for various reasons, decide to postpone motherhood, especially for women under 36 years of age.
